# Comprehensive Analysis of Neural Network Inference on Embedded Systems: Response Time, Calibration, and Model Optimisation †

**DOI:** 10.3390/s25154769

**Published:** 2025-08-02

**Authors:** Patrick Huber, Ulrich Göhner, Mario Trapp, Jonathan Zender, Rabea Lichtenberg

**Affiliations:** 1 Institute for Driver Assistance and Connected Mobility (IFM), Kempten University of Applied Sciences, Junkerstraße 1A, 87734 Benningen, Germany; 2 School of Computation, Information and Technology (CIT), Technical University of Munich, Boltzmannstraße 3, 85748 Garching, Germany; 3 Department of Informatics, Kempten University of Applied Sciences, Bahnhofstraße 61, 87435 Kempten, Germany; 4 Fraunhofer Institute for Cognitive Systems IKS, Hansastraße 32, 80686 Munich, Germany

**Keywords:** ANN inference, Tensorflow Lite, embedded systems, benchmarking, model calibration, response times

## Abstract

The response time of Artificial Neural Network (ANN) inference is critical in embedded systems processing sensor data close to the source. This is particularly important in applications such as predictive maintenance, which rely on timely state change predictions. This study enables estimation of model response times based on the underlying platform, highlighting the importance of benchmarking generic ANN applications on edge devices. We analyze the impact of network parameters, activation functions, and single- versus multi-threading on response times. Additionally, potential hardware-related influences, such as clock rate variances, are discussed. The results underline the complexity of task partitioning and scheduling strategies, stressing the need for precise parameter coordination to optimise performance across platforms. This study shows that cutting-edge frameworks do not necessarily perform the required operations automatically for all configurations, which may negatively impact performance. This paper further investigates the influence of network structure on model calibration, quantified using the Expected Calibration Error (ECE), and the limits of potential optimisation opportunities. It also examines the effects of model conversion to Tensorflow Lite (TFLite), highlighting the necessity of considering both performance and calibration when deploying models on embedded systems.

## 1. Introduction

The ongoing shift toward Industry 4.0 and an increasingly data-driven society significantly raises the demand for local preprocessing and intelligent evaluation of sensor data at the source on an edge device. As such, new applications for integration on edge devices become necessary. However, one challenge therein consists of the limited computational power (due to cost and energy efficiency) of edge devices [1]. Major ANN providers address these requirements by offering conversion of resource-intensive models into optimised ones, one example of which is the conversion from TensorFlow to Tensorflow Lite (TFLite) models.

Most existing benchmarking studies, however, focus on image classification tasks and evaluate performance using pre-trained convolutional networks like Residual Neural Network with 50 Layers (ResNet50), Visual Geometry Group from Oxford CNN (VGG16), or MobileNetV2 [2,3]. These works typically report performance in terms of frames per second, which reflect throughput requirements in computer vision pipelines.

Outside of that field, the focus is broadened (in signal processing), targeting the response times of processes. For example, in predictive maintenance, securing continual processing to predict changes in the system states is of particular interest. Overloading a system is to be avoided, while preemptive reactions should remain functional. This poses the question of which influencing factors can be used to determine the response times of processes utilising neural networks.

Despite extensive research on integrating deep learning into IoT systems such as the overview provided by [4], there is a lack of systematic analyses regarding the actual inference performance, calibration, and optimisation of models on embedded hardware. Therefore this work addresses two central challenges in deploying neural networks on embedded platforms:

1. The lack of systematic benchmarks for generic ANN architectures. While prior work predominantly benchmarked specific pre-trained models for vision tasks, we evaluate generic and application-independent networks on three different embedded systems. This systematic evaluation varies input/output dimensions, network depth, width, and activation functions to provide empirical insights into how these factors affect response time. These findings underscore the importance of lightweight and optimised models, which motivates our investigation into a second challenge.
2. Limited insight into the effects of model conversion on calibration. Using the ECE as a calibration metric, we investigate how the conversion of Keras models to TFLite impacts the reliability of predicted confidences. While accuracy preservation during conversion has been well studied, e.g., [5], the impact on calibration remains insufficiently addressed.

We differentiate our work from real-time capabilities, as the focus is on timely, but not necessarily immediate, predictions. We evaluate the ability of embedded systems to continuously process incoming signals without overload. While worst-case execution time (WCET) analysis is relevant in safety-critical real-time systems [6], many practical use cases are implemented on embedded platforms operating under a standard software configuration without real-time guarantees. In such environments, ensuring sustained throughput and avoiding data backlog is of greater practical importance than enforcing strict execution time bounds for each inference step. This work assumes that a model with sufficient accuracy has been identified for the task at hand, since our focus is on estimating and comparing response times across different embedded systems, based on the model’s architecture.

In addition to response times, the reliability of neural network predictions, particularly in terms of confidence calibration, is increasingly gaining importance in safety-critical or decision-relevant applications. Accurate confidence estimates allow systems to assess the trustworthiness of their outputs, which is essential for tasks such as anomaly detection or predictive maintenance. In this context, the calibration of ANN models becomes a relevant evaluation criterion alongside performance. In sensor-based systems, where decisions are made autonomously, miscalibrated models can cause overconfident predictions that lead to erroneous decisions or degraded system performance. Since predictive confidence closely correlates with classification correctness, our calibration results offer indirect but meaningful insights into accuracy preservation and can reveal effects that may not be captured by top-1 accuracy alone.

Section 2, Section 3, Section 4 and Section 5 focus on the analysis of response times in neural network inference on embedded systems. Section 2 reviews the network dimensions and problem complexity, categorising the chosen dimensions of the evaluated networks. Section 3 outlines the experimentation setup, followed by Section 4, which analyses the influence of network structure on response times. This analysis helps reduce experimentation parameters based on their relevance. Section 5 details the conducted experiments. Section 6 investigates the influence of network structure on model calibration, while Section 7 addresses the impact of converting the neural network models to TFLite on model calibration. Finally, the paper is concluded in Section 8.

## 2. Net Dimensions

### 2.1. Review of Net Dimensions and Problem Complexity

Classic image processing often utilises highly complex networks containing hundreds of thousands, if not millions, of weights in order to solve such complex problems. Sizing of the net dimensions for the experiments in this work is tailored to signal processing (e.g., predictive maintenance), however. Time series forecasting or anomaly detection as performed here usually do not require millions of weights [7,8,9].

For example, the paper [10] trained a Multi-Layer Perceptron (MLP) for predictive maintenance in substations, identifying eleven influencing factors that were evaluated using neural networks. These networks consisted of eleven input neurons, at most 20 layers, and one output neuron. The paper [7] forecasts the highest temperature to be expected in South Korea, evaluating multiple networks with between 49 and 1001 neurons for that prediction. Meanwhile, the paper [8] predicts the Key Performance Indicators (KPIs) of computers using a very simple neural network with five layers.

These examples demonstrate the relevance of such networks in real-world applications. Research proves that many networks with under 40,000 neurons provide accuracy balanced with performance within a range of 98% to 99.5% [9].

### 2.2. Sizing of the Experiments’ Net Dimensions

**Figure 1:**
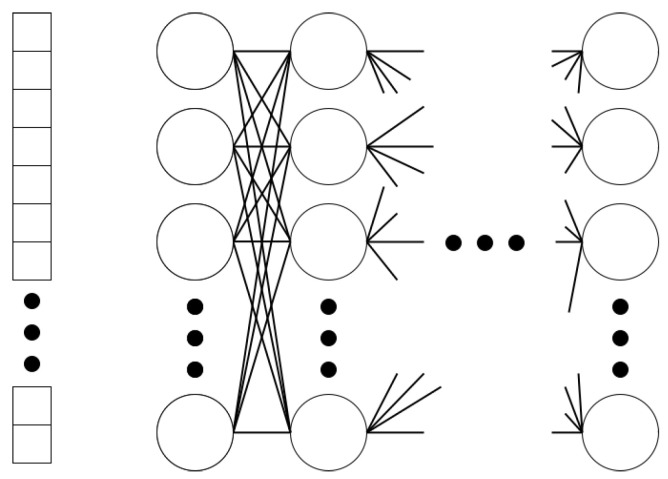
Rectangular neural network architecture with different input dimensions.

The networks chosen for the experiments are all equivalent in shape. All networks evaluated here are rectangular and fully connected. Their general architecture is pictured in Figure 1. In sizing the net dimensions for our experiments, we settled on a compromise between the aforementioned dimensions for image and signal processing from Section 2.1. This serves to ensure representation of the majority of signal processing applications by over-sizing.

The influencing factors of net dimensions encompass the number of layers, the number of neurons per layer, and the input and output dimensions, which vary in strides of ten each, as follows:

- Input/output dimensions between 1 and 91 (10 variations);
- Neurons per layer between 2 and 192 (20 variations);
- Layers between 2 and 192 (20 variations).

As a result, there are 4000 different network configurations, of which the most complex counts over seven million trainable weights, while the simplest has seven. The term layer herein covers input, output, and hidden layers. Subsequently, a network with two layers merely contains one input, one output, and no hidden layers.

## 3. Experimentation Setup

### 3.1. Time Measurement

To evaluate inference performance, we utilise wall time as the basis for measuring the duration of neural network execution. This approach ensures consistency between single- and multi-threaded execution scenarios, enabling proper comparison across different setups. In order to minimise potential distortion through interrupts, we perform 100,000 invocations with varying input vectors and calculate the mean duration of them, inspired by previous works [2,11]. Aside from executing the networks on one Central Processing Unit (CPU) core of the edge device, we also measured response times on multiple cores. However, we did not implement the multi-threading ourselves but initialised the TFLite inference with multiple threads enabled. Accordingly, throughout this work, we refer to response time, defined as the total time elapsed from invocation to output delivery, including computation, scheduling, and potential queuing overhead. This term is used in contrast to execution time, which refers strictly to the pure computational time for processing cores, and real time, which denotes compliance with fixed temporal deadlines [12]. Since embedded systems often operate under concurrent conditions and limited resources, the response time provides a more application-relevant metric for evaluating inference behaviour. It reflects the system-level performance as perceived by the application, which is critical for tasks such as continual sensor signal processing.

### 3.2. Dataset

The inference measurements conducted in this study use synthetic input data, as the focus lies on response times rather than classification accuracy. Regarding the learning process, input data influences only the trained weights of the network, not its architecture. Since the standard TensorFlow-to-TFLite conversion does not apply optimisations such as sparsity-aware execution or structured pruning [13], all weights—including zeros—are processed during inference, incurring the full computational costs. Execution follows a fixed, data-independent sequence of dense vector–matrix operations without dynamic control flow. This deterministic behaviour renders static WCET analysis methods based on control flow variability inapplicable. Instead, it mirrors unstructured pruning, where individual weights are masked but the network topology remains unchanged [14], leaving both the number of floating-point operations and the inference latency unaffected.

### 3.3. Evaluation Hardware

Benchmarking was performed on the systems listed in Table 1. All experiments were run on the internal CPU of the system. Hardware acceleration was omitted due to focusing on small networks, as its initialisation creates an overhead and produces additional costs for read/write operations on memory [15]. In addition, hardware accelerators such as the NPU of the 8MPLUSLPD4-EVK require quantification of the models since unsupported operations cause hopping between the CPU and accelerator, negatively impacting response times [15]. As the application of such quantifications affects the precision of the models, it stands in opposition to the goals set out in Section 3.2.

## 4. Analysis of the Influence of Net Dimensions and Structure

**Figure 2:**
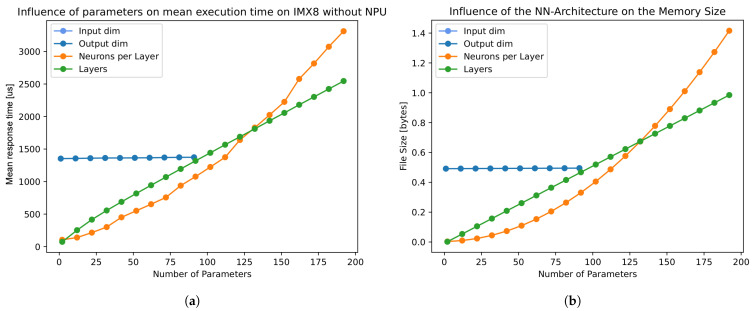
(**a**) Mean response times on IMX8 grouped by input dimension, neurons, and layers. (**b**) Corresponding memory usage.

This section inspects the influencing factors of the net dimension in accordance with Section 2.2 for response time and memory usage. Figure 2a shows the impact of each factor on mean response times, sorted by neurons per layer, layers, and input/output dimensions. It is noteworthy that input/output dimensions only marginally influence the mean response time over all variations. As the input and output dimensions overlap in this representation, merely the output dimension is evident. Additionally, we could prove through the experiments that the memory usage of the influencing factors has an equivalent impact on response times, as shown in Figure 2b.

The correlation between response times and memory usage implies dependence on the total amount of trainable parameters. Accordingly, Equations (1)–(4) are introduced to classify the results: Calculation of trainable parameters for the input layer $p_{il}$ (see Equation (1)) as well as the output layer $p_{ol}$ (see Equation (2)) is performed utilising the corresponding dimensions ($dim_{i}$ and $dim_{o}$) and the number of neurons *n*. Furthermore, the parameters of the hidden layers $p_{hl}$ are determined under consideration of the total amount of layers *l* (see Equation (3)). Subsequently, the sum of all parameters $p_{t}$ is calculated as shown in Equation (4).

(1) $$p_{il}=\left(dim_{i}+1\right)*n$$

(2) $$p_{ol}=\left(n+1\right)*dim_{o}$$

(3) $$\begin{array}{c} p_{hl}=\left[\left(n+1\right)*n\right]*\left(l-2\right) \end{array}$$

(4) $$p_{t}=p_{il}+p_{hl}+p_{ol}$$

For Equations (1)–(3), the bias of the previous layer is taken into account through incrementation. Subsequently, in accordance with Equation (3), there is a quadratic approximation of neurons per layer to the trainable parameters as well as a linear incline of layers, which aligns with the measurements shown in Figure 2. We found a linear correlation between the mean response times and total trainable parameters per network, indicating a strong dependence of these response times on the total parameters. Therefore, the empirical results (see Section 5.2) are presented in the form of a comparison between the two.

## 5. Conducting the Experiments

### 5.1. Experimentation Method

Empirical results are generated for the networks introduced in Section 2.2 on the chosen hardware platforms (see Section 3.3). In this section, the applied experimentation method complements the framework conditions of the existing experimentation setup outlined in Section 3, based on the previous findings concerning the influence of net dimensions and structure from Section 4. Accordingly, the framework conditions for the evaluation are defined as follows:

- As response times strongly depend on the total number of trainable parameters in a network as well as the hardware platform, these dimensions are compared.
- In order to reduce the amount of variation, networks are structured as rectangles because the number of trainable parameters and the response times of, e.g., pyramidal networks are enclosed in those of rectangular nets.
- For a further reduction in experiments, input and output layer variations were omitted and set to a constant of one due to the marginal influence on response times.
- Since a network consisting of twelve layers and two neurons per layer has the same number of trainable parameters (67 total) as one consisting of two layers and twenty-two neurons, networks with equivalent numbers of total trainable parameters are not measured anew.
- No processes aside from those necessary for the operating system were run concurrently with the benchmarking in order to minimise the impact of outliers (e.g., interrupts), and 100,000 calculations were run each.

**Figure 3:**
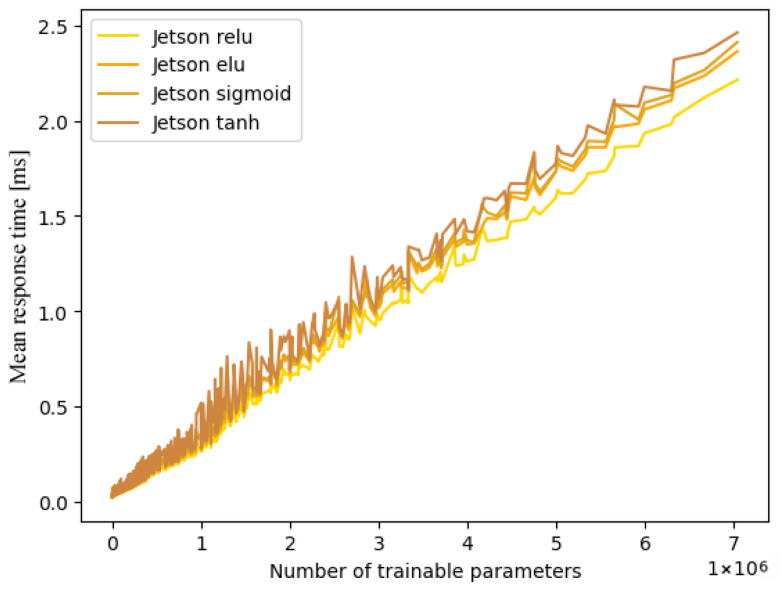
Comparison between activation functions on Jetson single core.

The initial experiments under these conditions proved the impact of varying activation functions and subsequent changes in calculation operations on response times, as expected. This behaviour is represented in Figure 3, displaying the single-thread performances on the Jetson architecture. Accordingly, one experiment was conducted for each of the most common activation functions: Rectified Linear Unit (ReLU), Exponential Linear Unit (ELU), Sigmoid and Tangens Hyperbolicus (TanH). Due to the linear correlation described in Section 4, and because displaying all measurement results would be unhelpful due to the extensive scope, our results are approximated via linearisation. Additionally, we provide the maximum deviation in ms in relation to the aforementioned linearisation. This procedure permits approximations for the given hardware platforms and activation functions outside of the chosen net dimensions (number of trainable parameters) by linear extrapolation. The precision of the measured data is of the order of nanoseconds, which is why the results are given in milliseconds with six decimal places.

### 5.2. Empirical Results

#### 5.2.1. Single-Threading

**Figure 4:**
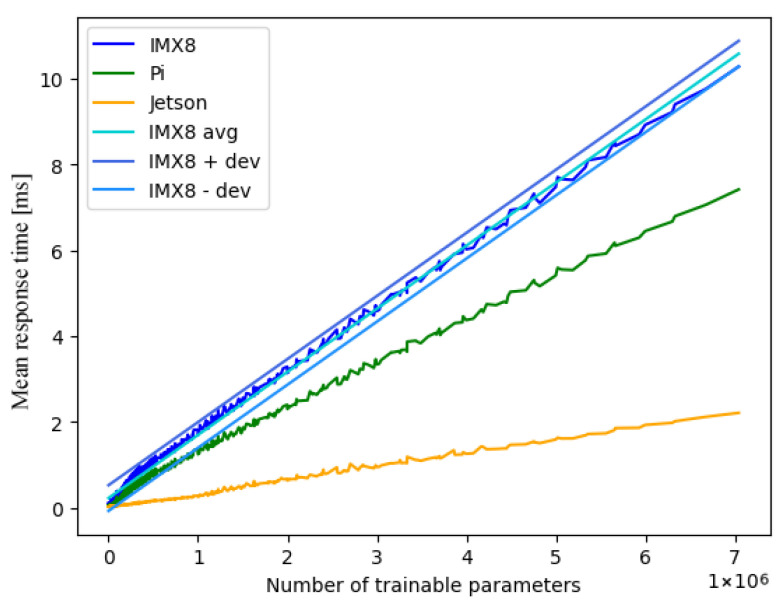
Single-thread ReLU performance across all architectures, including averages and deviations.

Figure 4 illustrates the classification of response times across the different hardware platforms. For improved readability, only the ReLU measurements are given. Additionally, this figure shows the linearisation for IMX8 via avg. Since the relation of response times to total trainable parameters has a linear trend, yet does not incline monotonously, we consider −dev as well as +dev to emphasise this fact. In addition, the corresponding absolute value of the maximum deviation in relation to the linearisation is provided.

Analysis of the empirical results, as shown in Table 2, shows minimal values for both the gradient and y-axis sections in the case of the ReLU activation function on the Jetson architecture, leading to minimal response times and, accordingly, the best performance.

The values for the gradient and y-axis sections imply an ascending order of activation functions in regard to response times across all hardware platforms as follows:

1. ReLU;
2. ELU;
3. Sigmoid;
4. TanH.

Additionally, the hardware platforms can be sorted with regard to response times for single-threading in ascending order, as shown in Table 3. The maximum deviations vary between around 0.15 ms and 0.55 ms. The values for the maximum deviation in Table 2 imply a reduction in deviation in the case of improved hardware performance; however, we deem the impact of activation functions on the deviations too insignificant to draw conclusions.

#### 5.2.2. Multi-Threading

**Figure 5:**
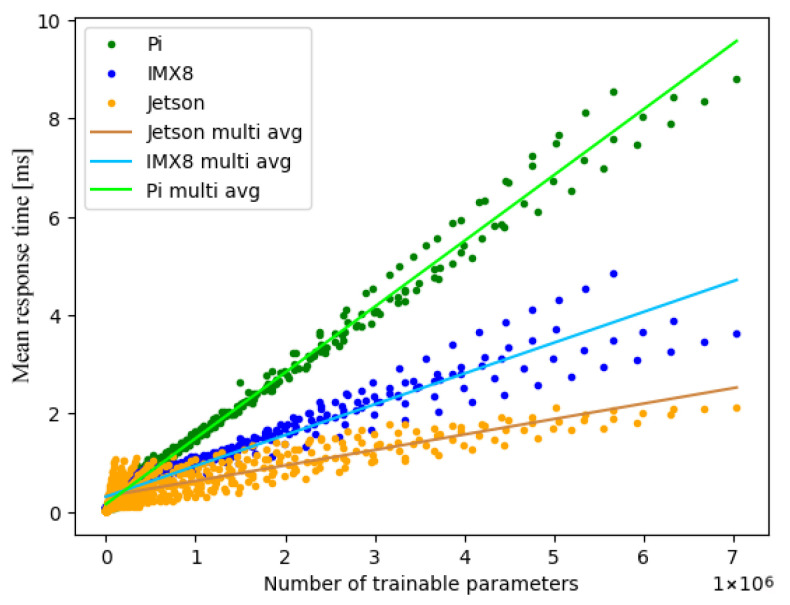
Multi-thread ReLU performance across all architectures, including averages.

The experiments were repeated for multi-threading, using four threads each for the sake of comparability. Figure 5 illustrates the classification of response times for the ReLU activation function across different hardware platforms. For the comparison between multi- and single-threading (see Section 5.3), we provide the linearisation via avg and maximum deviation like before.

It is noteworthy that the platforms IMX8 and Raspberry Pi generate linear groups of measurement results. Despite the fact that both architectures possess four CPUs each, the Raspberry Pi only generates three such prevalent lines, while the amount of lines is in accordance with the number of CPUs for IMX8. This behaviour indicates a possible difference in task scheduling; potential influencing factors for this are presented in Section 5.4. Analysis of the empirical results, as shown in Table 4, based on the gradient and y-axis sections shows minimal response times and, accordingly, the best performance for the ReLU activation function on the Jetson architecture.

The impact of the activation functions on response times generates differentiated behaviour in the case of the ELU and Sigmoid on Raspberry Pi. While a low total number of trainable parameters leads to a higher response time for the Sigmoid activation function compared to the ELU (see y-axis sections), this behaviour inverts upon increasing the total number of trainable parameters due to the low gradient inclination. Aside from the aforementioned differential behaviour, the measured values imply equivalent sorting of activation functions in regards to response, times as seen in single-threading (Section 5.2.1).

Additionally, the hardware platforms can be sorted with regard to the response times according to Table 3 for multi-threading. The maximum deviations vary between around 0.72 ms and 2.85 ms. Analysis of the deviations shows no significant correlation with platform performance, meaning that the scattering does not necessarily align with the platform’s potency. Deviations are minimal for the ReLU activation function on all platforms, while the TanH function always generates the greatest deviations. The maximum deviations correlate with the net dimensions for IMX8 and Raspberry Pi, meaning greater scattering for larger networks. Due to the aforementioned forming of lines, we propose the hypothesis that this is caused by different task partitioning and scheduling strategies. For this reason, we will take a closer look at this line formation in Section 5.4.

### 5.3. Comparison Between Multi- and Single-Threading

The empirical results show differentiated behaviour with regard to the deviation when comparing multi- and single-threading. There exists a general increase in scattering for multi-threading when compared to single-threading. Additionally, scattering is also influenced by the hardware platform, dependant on the total number of trainable parameters. This varying behaviour becomes evident in the comparison of deviations on IMX8 and Jetson, as shown in Figure 5. In order to maintain clarity for the multitude of variations, we retained the measure for the deviation previously introduced in Section 5.1. Therefore, when using linearisation as extrapolation, the deviation values are undetermined for multi-threading in contrast to single-threading.

Different activation functions generate differentiated scattering as well, despite running on the same hardware. For example, the maximum deviation varies between 0.72 ms (ReLU) and 2.85 ms (TanH) for multi-threading on Jetson, while single-threading varies far less, between 0.15 ms (ReLU) and 0.27 ms (TanH). However, the response times showed unexpected behaviour. Only the IMX8 architecture wholly reduced response times through multi-threading, as expected. Meanwhile, against our expectations, Jetson generated a noticeable increase in the gradient by about 183.24% for the TanH function while also increasing the y-axis section value, resulting in delayed response times. The variations in response times for the remaining activation functions on Jetson were lesser, yet they implied ineffective task partitioning and scheduling for multi-threading when compared to single-threading.

The Raspberry Pi consistently produced higher response times across all activation functions when using multi-threading. In addition to the worsening of response times, the further reduction on IMX8 causes it to overtake the Raspberry Pi for multi-threading, as seen in Table 3. Taking the CPU benchmark for the integrated processors into account, wherein the ARM Cortex-A72 4 Core (Raspberry Pi) outperformed the ARM Cortex-A53 4 Core (IMX8) [16], the user would not expect such behaviour.

**Figure 6:**
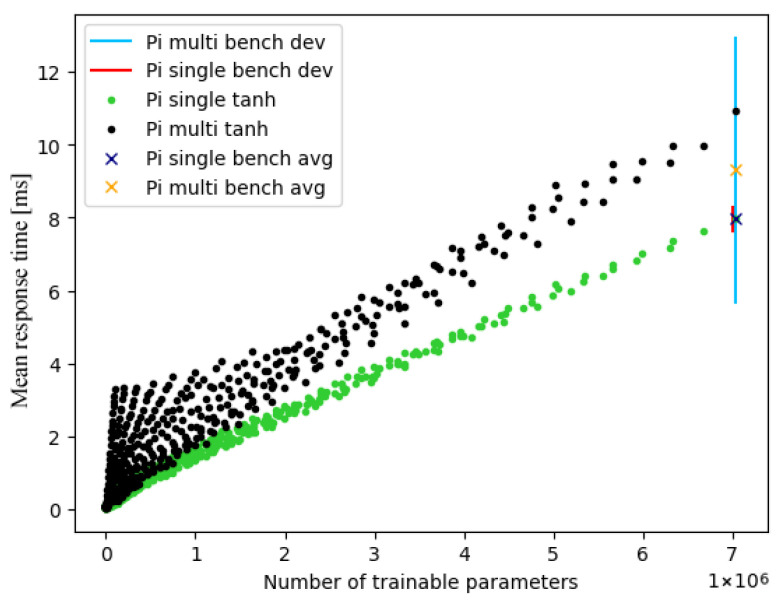
Raspberry Pi TanH performance on single- and multi-threading compared to average and deviation obtained from library benchmarking tool using largest model.

In order to eliminate the possibility of systematic errors on our end, we chose to compare our data with an alternative benchmarking tool, for which the onboard TFLite benchmark tool was utilised. This tool is tailored to producing empirical results that are as exact as possible for any given model. To this end, for example, specific warm-up invokes are performed in advance [17]. However, it is not suited to measuring many varying models, as was our use case. Figure 6 shows the comparison of behaviours for the Raspberry Pi using the TanH activation function. Additionally, dev and avg of the TFLite benchmark tool (referred to as bench) were included, measured for the largest model. Ultimately, the tool supports our findings as its measurements generate a significant increase in response times in the case of multi-threading compared to the single-thread execution as well. As was the case for our measurements, the tool’s measured deviation increased for multiple cores compared to running on just one.

### 5.4. Side Effects

In the previous sections, it was proven that both the hardware platform as well as the activation function cause differentiated behaviour with regard to response times. Additionally, the choice of threading partially had an unexpected impact (see, e.g., the Raspberry Pi in Section 5.2.2). Subsequently, further potential side effects were analysed during the experiments. Logging of the tact rate eliminated the possibility of the minimum and maximum tact rate span causing variations in response times. Furthermore, we had the hypothesis that the simultaneous use of multiple cores could result in changes in system temperature. This is potentially supported by the different cooling systems. When comparing the tact rates of the ARM Cortex-A72 4 Core (Raspberry Pi) and ARM Cortex-A53 4 Core (IMX8), as seen in Table 5, at first, one might think they imply an explanation for the worsening of response times, but our logs showed no sign of tact rate throttling during the experiments. Furthermore, the logs did not reveal any RAM bottlenecks that could have led to increased page faults.

Subsequently, the evident increase in response times in multi-threading implies partially inefficient task partitioning and scheduling. These could facilitate a pipeline hazard, potentially further increasing response times in addition to the scheduling overhead. Due to the heterogeneous hardware architectures with regard to, e.g., caching, individual identification of influencing factors requires detailed inspections well beyond the frame of this work. While the TFLite documentation mainly attributes multi-thread performance variability to concurrently running applications, our results show that such effects also occur under controlled conditions without additional user processes [18]. This suggests that the variability is inherent to the platform and the TFLite runtime and not solely caused by external interference.

**Figure 7:**
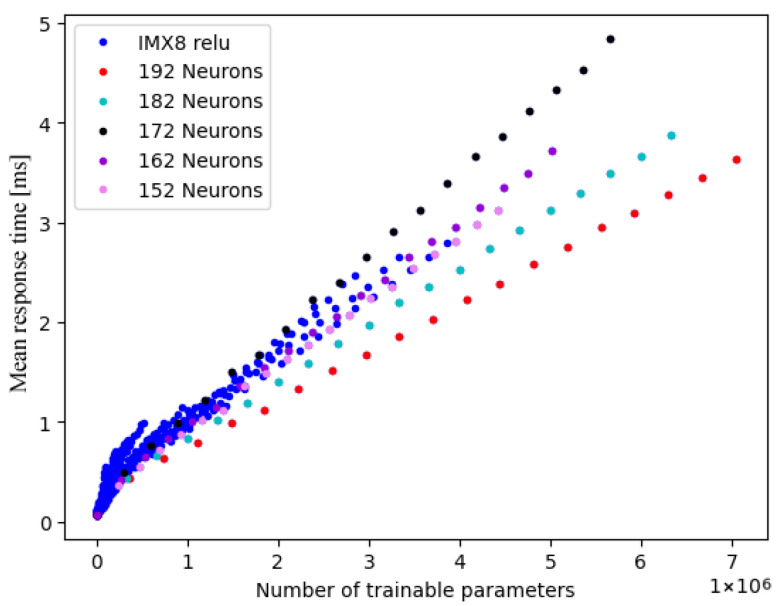
IMX8 performance on multiple threads with selected individual lines based on neurons.

During our inspection of the results, we managed to select individual lines from the multi-threading plots from the number of neurons per layer (see Figure 7). This further supports our hypothesis concerning inefficient task partitioning and scheduling, as the network structure is one of its influencing factors. This is illustrated by the fact that there is no direct correlation between the number of neurons and the reaction time. However, there is a linear trend for any number of neurons per layer. In conclusion, it is important to remember that the optimal number of threads depends on a multitude of factors like the means of calculation, the CPU architecture, the type of model, and the available resources.

## 6. Impact of the Network Structure on the Model Calibration

Previously, we investigated the influence of net dimensions (size) in relation to (thus) solvable problem complexities (cf. Section 2.1) and showed the impact of the network structure (cf. Section 4) and number of trainable parameters on response times through our experiments (cf. Section 5). Problem manageability, accuracy, and response times are generally comprehensive factors for ANN users and are consequently a primary focus when choosing the network’s architecture and size.

(5) $$\mathrm{ECE}=\sum_{m=1}^{M}\frac{|B_{m}|}{n}\cdot \left|\mathrm{acc}\left(B_{m}\right)-\mathrm{conf}\left(B_{m}\right)\right|$$

However, the influence of net dimensions on the model’s calibration is less intuitive. Measuring the calibration error can be achieved, for example, through the Expected Calibration Error (ECE), as calculated in Equation (5) [19]. The error for each confidence interval $B_{m}$ (bin) is computed as the absolute difference between the accuracy acc and the average confidence conf. This confidence, or the underlying logits, is used as the basis of the classification result, for example, via the argmax operator [20]. If calibration is not considered and increasing uncertainties arise in the real world due to changing environmental conditions (distribution shift), the confidence and, consequently, the classification result will no longer be representative. This may result in an unreliable classifier, which would no longer produce robust results.

Hence, a calibration that accurately represents the possibility of erroneous classification and provides a realistic assessment of the results reliability is pursued [19]. For this reason, this section examines the controversy in the existing literature regarding whether improved calibration necessarily requires larger networks or whether it can potentially be achieved with smaller networks through targeted pruning. Some studies suggest that scaling the model size itself has a positive impact on calibration. For example, as described in [21], “Generally, larger models produce better calibrated results while the level of such effect is diverse among tasks”. One possible explanation for this behaviour could be that larger models, due to their higher capacity, are better suited to capture the underlying data distribution. These results contrast with previous works, which show that larger networks increase accuracy but tend to become overconfident, leading to deterioration of the ECE. It is also pointed out that these deteriorations occur in all the studied network architectures, leading to the conclusion that this issue is not architecture-specific [19]. At the same time, improvements to greater-net-dimension models could be achieved through the use of calibration techniques (such as temperature scaling). However, it has not been quantified whether such optimised models can provide equally good calibration results for the same tasks as smaller networks [19].

More recent comparisons of calibration behaviour between variously sized models within one model family outline a trend: At first, calibration deteriorates with increased size. However, this effect inverts with increased distribution shift—moreso when temperature scaling is additionally applied for the optimisation of calibration. As summarised in [22], “the calibration of larger models is more robust to distribution shift”. In addition, it has been found that newer model architectures exhibit less pronounced deterioration of calibration with increasing model size [22]. This disproves the previous hypothesis concerning the lack of influence of underlying architectures on calibration (cf. [19]).

Artificial Neural Networks (ANNs) are defined by their architecture, from which the number of underlying parameters (net dimension) can be derived. The comprehension depth such a model can develop, and subsequently, how fit it is to solve a specific task, depends on the architecture and resulting net dimensions [20].

A deeper understanding of the data distribution can help to identify better features for the model and improve generalisation, provided this is possible for the specific task and data foundation.

We refer to the ability to detect a new trait as a (new) cognitive step. Further, let it be considered that a model might hold additional capacities, meaning a greater net dimension than strictly necessary for solving the current problem. These additional capacities persist until the next cognitive step is taken, i.e., the recognition of a more complex problem, if such a problem exists. We hypothesise that increased model capacity is utilised to amplify classification confidence through heightened activation levels, especially in the absence of calibration regularisation.

As described in [19], once a model has learned to carry out classification correctly, the negative log likelihood (NLL) can be further minimised by increasing the confidence of its predictions, thereby leading to overconfidence. This hypothesis is further supported by the following claim [19]: “Though we cannot claim causality, we find that increased model capacity and lack of regularisation are closely related to model miscalibration.” Subsequently, overconfident cases need to be regulated. According to our hypothesis, parameters should be removable up to the point where a cognitive step is lost in order to reduce overconfidence while maintaining accuracy. This should allow for an improvement in calibration through pruning.

Our research found existing works that have inspected the impact of state-of-the-art post hoc pruning methods on calibration and robustness [23]. There are also approaches that, for example, analyse the uncertainty of model weights during training through magnitude-based pruning [24]. The results of these works support our hypothesis that pruning holds the potential to improve model calibration [23,24]. Regarding predictive power, the optimisations in [24] resulted in merely small losses.

Previous results underline the relevance of reducing trainable parameters, especially in the area of embedded applications. However, it is essential to not only consider accuracy and response times but also calibration error in order to preserve model quality. This is particularly critical in sensor-driven systems with safety or fault detection applications, where overconfident misclassifications may lead to missed anomalies or false alarms. Our literature review indicates that these objectives are not mutually exclusive. We aim to increase awareness of the importance of calibration in order to enable more applications to produce robust results in real scenarios.

## 7. Impact of Conversion to TFLite on Model Calibration

As outlined in the context of this work, model inference in many practical scenarios, such as mobile applications or embedded systems, does not take place within the original training environment. Instead, it is performed on optimised platforms such as TFLite. Whether and to what extent the calibration properties are affected in this context have, to date, been insufficiently investigated. In the realm of embedded sensor systems, where TFLite models are widely deployed due to their efficiency, this knowledge gap is particularly impactful. We therefore address the question of how the conversion of a neural model into the TFLite format affects its model calibration, as measured by the ECE. The aim is to gain empirical insights into whether the conversion may lead to a loss in model reliability, even when the top-1 accuracy is nominally preserved.

### 7.1. Model and Dataset Selection

For the systematic selection of suitable models and datasets, we consider the following criteria:

- Relevancy to current research;
- Availability and reproducibility;
- TFLite compatibility;
- Comparability to existing benchmarks.

#### 7.1.1. Use Case: Image Recognition

The Vision Transformer model ViT-B/16 (Base, patch size 16) represents a member of a more recent class of architectures based on self-attention mechanisms. Since its publication, ViT-B/16 has established itself as a baseline model in research [22,25]. Our experiments were conducted using the ViT-B/16 model from the ViT Keras package, pre-trained on ImageNet 2012 [26] and evaluated on its validation dataset, in line with previous work [22,27]. The model comprises approximately 86.9 million trainable parameters, reflecting its substantial capacity compared to lightweight models. As we do not intend to apply additional calibration techniques, we make use of the entire validation dataset. ImageNet 2012, as an established standard dataset, ensures a high degree of comparability with existing studies and, due to its scale, helps to ensure that observed effects cannot be attributed to the characteristics of small or simple datasets. To the best of our knowledge, this study provides the first empirical assessment of the calibration robustness of Transformer architectures under conversion to TFLite. The results offer insights into the suitability of modern models for edge deployment and inform whether post-conversion calibration steps (e.g., temperature scaling) are necessary [19,22]. The calibration analysis of the image recognition model in the context of this work is to be understood as an over-sizing example, following the approach outlined in Section 2.2.

#### 7.1.2. Use Case: Signal Processing

Therefore, as a second use case, we investigate a practical natural language processing application. The goal is to extend the study, within the context of this work, by exploring a lightweight model for signal processing. In the interest of reproducibility, we use the publicly available TFLite Speech Recognition demo [28]. The methodology employed is based on a signal processing-typical Mel-Frequency Cepstral Coefficient (MFCC) preprocessing step, combined with a convolutional neural network model. This represents a typical example of modern signal processing applications in deep learning, where the boundaries between classical signal processing and visual classification are increasingly blurred. The model was specifically designed for TFLite contexts and is therefore highly compatible. It is lightweight, comprising fewer than 17,000 trainable parameters, making it well-suited for deployment on resource-constrained devices. For the evaluation of calibration properties, we use the Speech Commands v2 test dataset [29].

### 7.2. Empirical Results

#### 7.2.1. Use Case: Image Recognition

First, we analyse the distribution of samples across the predicted confidences using the confidence histogram, in order to highlight the bins in which the model predominantly operates. This enables a well-founded interpretation of the weighting of individual bins in the context of the ECE. Further comparison of the reliability diagrams allows for a quantitative assessment of the impact of conversion on the model’s calibration.

**Figure 8:**
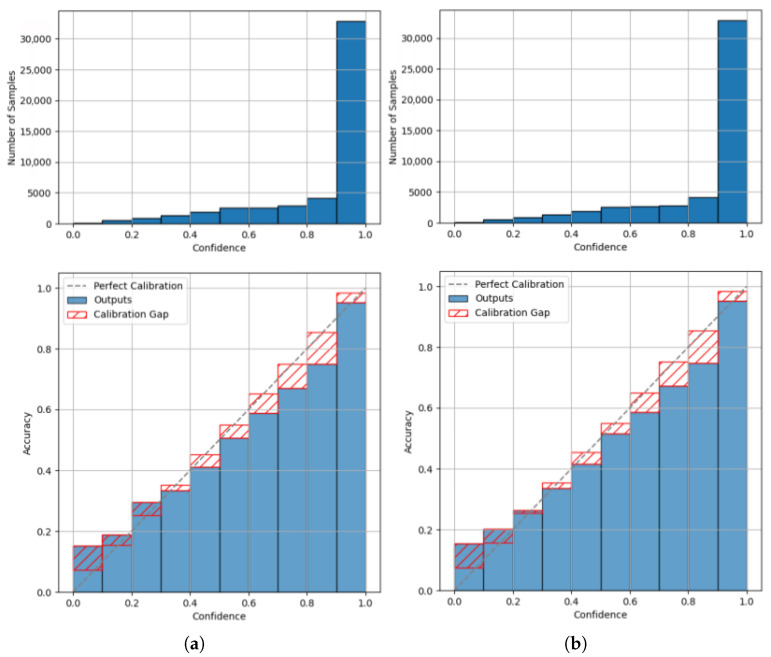
Confidence histogram (**top**) and reliability diagram (**bottom**) for the Keras model (**a**) and the TFLite model (**b**) in the image recognition use case.

A comparison of the plots for the Keras base model (Figure 8a) with those of the converted TFLite model (Figure 8b) reveals that there are only marginal deviations in both the confidence distribution and the reliability diagrams. One example of such a deviation can be observed in the reliability diagrams in the interval (0.2–0.3]. Qualitatively, when considering the ECE for 15 bins, the Keras model yields an ECE of 0.0431, while the TFLite model shows an ECE of 0.0420. This indicates a marginal improvement in calibration due to the conversion. Given the known sensitivity of the ECE to the number of bins, we also conducted the experiments using 30 bins to rule out insufficient resolution as a cause. Subsequently, we obtained an ECE of 0.0433 for the Keras model and 0.0426 for the TFLite model, confirming a slight variation in score but no change in the overall interpretation.

#### 7.2.2. Use Case: Signal Processing

**Figure 9:**
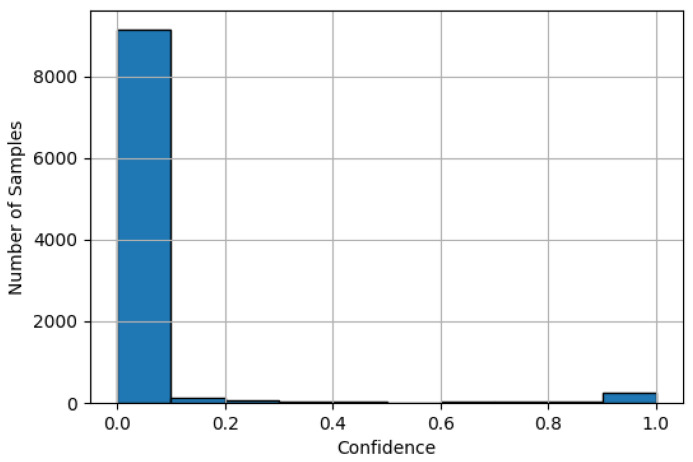
Distribution of sigmoid-based confidences for the target class.

In the signal processing use case, only one wake word is detected, classifying it as a binary classification problem. In binary classification problems, the method for determining the average accuracy used for ECE calculation differs. In multi-class classification, this average accuracy is derived from the top-1 accuracy, which compares the model’s prediction with the ground truth label. In contrast, for binary classification, the empirical accuracy is used, which, for each confidence interval, solely considers the ground truth labels and compares how frequently the desired class is present [30,31]. Similarly, the determination of the confidence differs: in binary classification, the confidence is predicted for a single class, typically (and in our case) using a sigmoid function. This contrasts with multi-class problems, where the prediction is made as a confidence distribution over all classes, typically using a softmax function. Figure 9 shows the distribution of sigmoid activation values for the target class. The plot indicates a high concentration of negative class samples, particularly in the lower confidence range (0.0–0.1]. The data in Figure 9 are based on the outputs of the Keras model. Due to scaling, deviations compared to the TFLite model are not visually discernible, and therefore, separate plots for the TFLite model are omitted.

**Figure 10:**
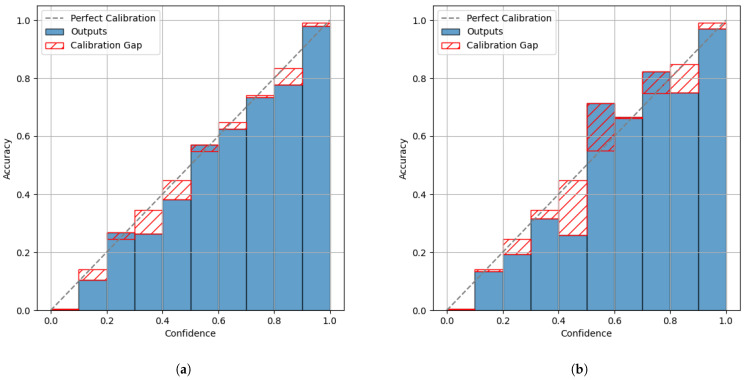
Reliability diagram for the Keras model (**a**) and the TFLite model (**b**) in the signal processing use case.

The comparison of the reliability diagrams (see Figure 10a,b) quantitatively suggests a degradation in calibration after conversion to TFLite. However, with an ECE of 0.0041 for the Keras model and 0.0042 for the TFLite model (using 15 bins), this difference is barely captured numerically. This mismatch between quantitative and qualitative analyses reflects ECE’s limited sensitivity to local calibration effects.

**Figure 11:**
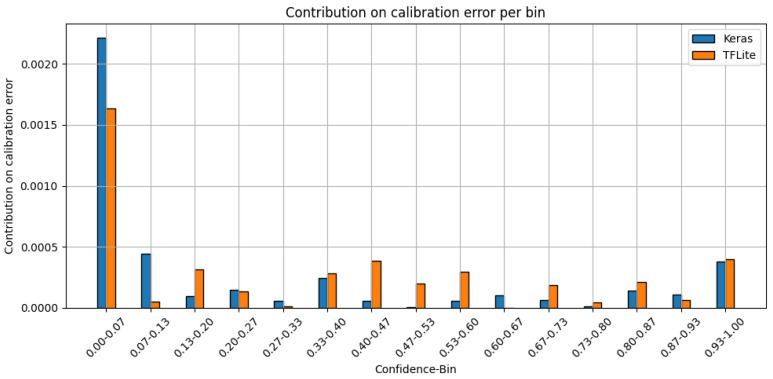
Per-bin contribution to the ECE for the signal processing use case (15 bins), highlighting the individual impact of each confidence bin on the overall calibration error.

To enable a more detailed analysis of calibration quality, Figure 11 shows the per-bin contribution to the ECE. The per-bin calibration error is defined as the absolute difference between the average confidence and the accuracy within each bin, weighted by the proportion of samples falling into that bin. This reveals that the majority of the calibration error occurs within the interval (0.0-0.1], which is due to the previously mentioned concentration of negative class samples falling into this bin (cf. Figure 9). Even a small deviation per sample leads to a significant overall contribution to the total calibration error due to the bin’s weighting.

**Figure 12:**
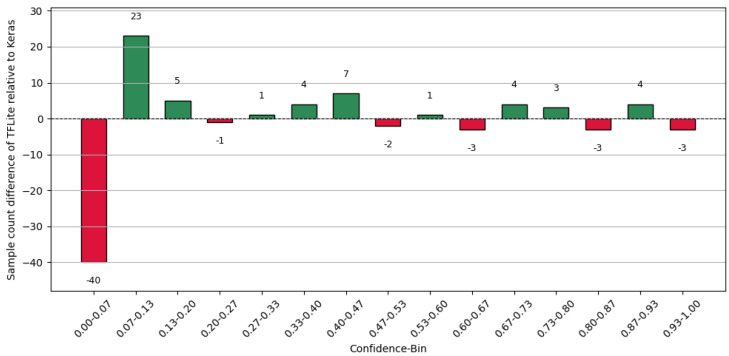
Difference in the number of samples per bin (TFLite relative to Keras) for the signal processing use case (15 bins).

Furthermore, the conversion of the Keras model to TFLite results in a shift in the confidences for individual samples, depending on the chosen granularity of the confidence intervals (number of bins), as shown in Figure 12. This redistribution of samples consequently affects the mean confidence and accuracy used to compute the ECE for each respective bin. As a result of the presented experiments, the conversion of the Keras models to TFLite leads to a marginal variation in the ECE, caused by a shift in the confidences, which is reflected in the ECE depending on the chosen resolution (number of bins). This underlines a known limitation of the ECE: its reliance on bin-averaged aggregation may obscure finer-grained calibration differences [32].

## 8. Conclusions

This study presents a systematic evaluation of the inference response times and calibration behaviour of ANNs on embedded platforms. Using fully connected networks with varying architectures and activation functions, we analysed the influence of network structure, hardware platform, and threading strategy on response times. The empirical data permitted approximation of response times for ANN models on the chosen platforms, which in turn allow the user to configure their model for continual stream processing. As such, this paper answers an unmet demand in benchmark research by extending the focus from existing image processing networks to generic ones.

Rather than exploring task-specific accuracy trade-offs, we assume that a suitable model has already been selected. Our aim is to enable users to estimate the response time of such models under realistic conditions and compare inference behaviour across embedded systems. Additionally, we quantify the impact of model conversion (Keras to TensorFlow Lite) on calibration quality using the ECE.

### 8.1. Lessons Learned

Our experiments reveal the following insights:

1. Activation functions influence thread-level performance. Beyond arithmetic complexity, different activation functions affect how efficiently computations scale under multi-threaded execution. ReLU consistently shows stable performance, whereas TanH suffers from degraded parallel efficiency—suggesting less favourable interaction with the runtime scheduling (cf. Section 5.2).
2. Multi-threading behaviour is strongly platform-dependent. Contrary to expectations, multi-threading sometimes leads to significantly higher response times instead of improvements. This unexpected degradation points to inefficiencies in current partitioning and scheduling strategies of state-of-the-art libraries, highlighting an urgent need for optimisation tailored to specific hardware and model characteristics.
  - IMX8 profits from thread-level parallelism;
  - Raspberry Pi exhibits performance degradation under multi-threaded execution;
  - Jetson reacts variably depending on model configuration (Section 5.2.2 and Section 5.3).
3. Thread scheduling behaviour lacks transparency. Identical models yield inconsistent results across platforms despite uniform conditions. These effects indicate a complex interaction between runtime-level scheduling and model structure that is not visible or controllable at the user level (Section 5.4).
4. TFLite model conversion preserves calibration globally but alters local confidence patterns. Post-conversion evaluation is recommended, particularly for applications relying on confidence-based decisions (Section 7). This applies especially to systems where sensors act as autonomous decision triggers in real-world environments.

### 8.2. Outlook

Building on these findings, we propose the following directions for future work:

- Targeted use of pruning for calibration improvement. Prior work suggests that larger networks tend to be overconfident. Based on this, we hypothesise that systematical pruning may help regularise confidence by removing such overconfident subnets. Future work could explore pruning strategies optimised for calibration error, enabling smaller and better-calibrated models for embedded deployment.
- Extension to other model types. To assess the structural generalisability of response time modelling, model types that have not been presented in this work, such as recurrent or attention-based networks, offer themselves to further study.
- Development of adaptive scheduling mechanisms. Our analysis shows that the effectiveness of multi-threaded execution varies with model and hardware characteristics. To address this, future frameworks could monitor runtime behaviour and dynamically adjust scheduling strategies when inefficiencies are detected, improving inference performance under changing conditions.
- Reverse-engineering runtime scheduling via algorithm pattern detection. Due to limited transparency in TFLite’s multi-threading, future work could apply automated algorithmic pattern recognition to source code and runtime data [33]. This would help to uncover scheduling structures and dependencies, revealing bottlenecks and guiding optimisations.

In conclusion, this study adds to our understanding of the challenges that are tied to the utilisation of ANN models on embedded platforms with limited resources. Since demand for ANN applications for data sources keeps increasing, our findings may facilitate making well-founded decisions for reaching optimal performance in various real-world scenarios, balancing both efficiency and robustness.

## Figures and Tables

**Table 1:** Benchmarking hardware.

Name	NXP	Raspberry	NVIDIA
	8MPLUSLPD4-EVK	Pi 4 Model B	Jetson AGX XAVIER
Processor	ARM Cortex-A53 4 Core	ARM Cortex-A72 4 Core	NVIDIA Carmel ARM 8 Core
Clock Speed	1.80 GHz	1.50 GHz	2.20 GHz
Operating System	Yocto 5.15 (kirkstone)	Debian 11 (bullseye)	Ubuntu 20.04.6 (focal)
Manufacturer	NXP Semiconductors	Raspberry Pi Ltd.	NVIDIA Corp.
City, Country	Eindhoven, NL	Cambridge, UK	Santa Clara, CA, USA

**Table 2:** Gradients, y-axis sections, and maximum deviation coefficients for all activation functions across all architectures in single-threading.

Hardware	Activation	Gradient	Y-Axis	Maximum
Platform	Function		Section	Deviation
IMX8	ReLU	1.469174 × 10^−6^	2.303959 × 10^−1^	0.298859
	ELU	1.520086 × 10^−6^	3.118073 × 10^−1^	0.365074
	Sigmoid	1.524141 × 10^−6^	3.135349 × 10^−1^	0.378036
	Tanh	1.649902 × 10^−6^	4.050808 × 10^−1^	0.553396
Raspberry Pi	ReLU	1.071348 × 10^−6^	1.578266 × 10^−1^	0.286284
	ELU	1.112801 × 10^−6^	1.998119 × 10^−1^	0.345382
	Sigmoid	1.114504 × 10^−6^	2.092044 × 10^−1^	0.326310
	Tanh	1.151102 × 10^−6^	2.668625 × 10^−1^	0.392679
Jetson	ReLU	3.188076 × 10^−7^	1.063447 × 10^−2^	0.153478
	ELU	3.409927 × 10^−7^	2.067891 × 10^−2^	0.192275
	Sigmoid	3.475815 × 10^−7^	2.299473 × 10^−2^	0.164944
	Tanh	3.579973 × 10^−7^	4.256916 × 10^−2^	0.274846

**Table 3:** Ranking of hardware platforms for single- and multi-threading based on response times in ascending order.

Rank	Single-Threading	Multi-Threading
1	Jetson	Jetson
2	Raspberry Pi	IMX8
3	IMX8	Raspberry Pi

**Table 4:** Gradients, y-axis sections, and maximum deviation coefficients for all activation functions across all architectures in multi-threading.

Hardware	Activation	Gradient	Y-Axis	Maximum
Platform	Function		Section	Deviation
IMX8	ReLU	6.260788 × 10^−7^	3.092691 × 10^−1^	1.075668
	ELU	6.764540 × 10^−7^	3.874742 × 10^−1^	1.168902
	Sigmoid	6.788531 × 10^−7^	3.905559 × 10^−1^	1.129849
	Tanh	9.248984 × 10^−7^	1.003794 × 10^0^	1.746782
Raspberry Pi	ReLU	1.337886 × 10^−6^	1.586471 × 10^−1^	0.815641
	ELU	1.415562 × 10^−6^	2.039669 × 10^−1^	1.476009
	Sigmoid	1.399550 × 10^−6^	2.106196 × 10^−1^	1.007297
	Tanh	1.450218 × 10^−6^	9.194122 × 10^−1^	2.250828
Jetson	ReLU	3.143797 × 10^−7^	3.164306 × 10^−1^	0.715131
	ELU	3.473014 × 10^−7^	3.510930 × 10^−1^	0.769705
	Sigmoid	3.550585 × 10^−7^	3.552569 × 10^−1^	0.814380
	Tanh	6.556785 × 10^−7^	1.190182 × 10^0^	2.846824

**Table 5:** Tact rates as measured by lscpu command.

	Cortex-A72	Cortex-A53
CPU max MHz	1500	1800
CPU min MHz	600	1200

## Data Availability

The original contributions presented in this study are included in the article. Further inquiries can be directed to the corresponding author.

## Abbreviations

ANN	Artificial Neural Network
CPU	Central Processing Unit
CSV	Comma-Separated Value
ECE	Expected Calibration Error
ELU	Exponential Linear Unit
KPI	Key Performance Indicators
LLM	Large Language Models
MLP	Multi Layer Perceptron
MFCC	Mel-Frequency Cepstral Coefficients
NLL	Negative log likelihood
NPU	Neural Processing Unit
ReLU	Rectified Linear Unit
ResNet50	Residual Neural Network with 50 Layers
TanH	Tangens Hyperbolicus
TFLite	Tensorflow Lite
VGG16	Visual Geometry Group from Oxford CNN
WCET	worst-case execution time
